# A quality grade classification method for fresh tea leaves based on an improved YOLOv8x-SPPCSPC-CBAM model

**DOI:** 10.1038/s41598-024-54389-y

**Published:** 2024-02-20

**Authors:** Xiu’yan Zhao, Yu’xiang He, Hong’tao Zhang, Zhao’tang Ding, Chang’an Zhou, Kai’xing Zhang

**Affiliations:** 1 College of Information Science and Engineering, Shandong Agricultural University, Taian, China; 2 College of Mechanical and Electronic Engineering, Shandong Agricultural University, Taian, China; 3 Tea Research Institute, Shandong Academy of Agricultural Sciences, Jinan, China

**Keywords:** Fresh tea leaves, Grade discrimination, Target detection, Improve YOLOv8x, CBAM, Plant sciences, Computational models, Image processing

## Abstract

In light of the prevalent issues concerning the mechanical grading of fresh tea leaves, characterized by high damage rates and poor accuracy, as well as the limited grading precision through the integration of machine vision and machine learning (ML) algorithms, this study presents an innovative approach for classifying the quality grade of fresh tea leaves. This approach leverages an integration of image recognition and deep learning (DL) algorithm to accurately classify tea leaves’ grades by identifying distinct bud and leaf combinations. The method begins by acquiring separate images of orderly scattered and randomly stacked fresh tea leaves. These images undergo data augmentation techniques, such as rotation, flipping, and contrast adjustment, to form the scattered and stacked tea leaves datasets. Subsequently, the YOLOv8x model was enhanced by Space pyramid pooling improvements (SPPCSPC) and the concentration-based attention module (CBAM). The established YOLOv8x-SPPCSPC-CBAM model is evaluated by comparing it with popular DL models, including Faster R-CNN, YOLOv5x, and YOLOv8x. The experimental findings reveal that the YOLOv8x-SPPCSPC-CBAM model delivers the most impressive results. For the scattered tea leaves, the mean average precision, precision, recall, and number of images processed per second rates of 98.2%, 95.8%, 96.7%, and 2.77, respectively, while for stacked tea leaves, they are 99.1%, 99.1%, 97.7% and 2.35, respectively. This study provides a robust framework for accurately classifying the quality grade of fresh tea leaves.

## Introduction

Tea, renowned as one of the world's most cherished beverages, not only delights the palate but also boasts a wealth of beneficial nutrients, including catechins and anthocyanins, recognized for their potential in disease prevention. With the swift advancement of the economy, people are becoming increasingly health-conscious while enjoying improved living standards. This has led to a surging demand for tea, particularly the high-quality variety renowned for its rich nutrient content. Notably, despite high-quality tea only accounting for less than 5% of total tea production, it contributes to over 20% of the total value generated by the entire tea industry. Consequently, high-quality tea can generate substantial value^1^. However, a prevailing challenge in the tea industry lies in the harvesting and procurement of fresh tea leaves, which often consist of varying quantities of buds and leaves. This results in a blend of different grades of fresh tea leaves. These various grades exhibit differing levels of tenderness, and utilizing uniform processing parameters, such as the temperature, duration, frequency, and others of the withering process, can significantly damage the nutritional components and cause a decline in tea quality. Hence, it becomes imperative to classify fresh tea leaves prior to processing and to employ tailored processing parameters for each grade of fresh tea leaves. This can minimize the deterioration of nutritional constituents and enhance tea quality. Therefore, the development of a highly accurate grading method for fresh tea leaves carries significant importance in meeting the demands of the tea industry.

Presently, both domestically and internationally, tea leaves’ grading primarily employs two main methods: machine sorting and machine vision-based classification. Wang et al.^2^ introduced a grading machine designed for machine-harvested tea leaves, which segregates impurities, such as tea stems, from fresh tea leaves via air sorter and subsequently categorizes the tea leaves by belt screening. Lv et al.^3^ developed a vibration grading machine specially designed for quick and effective grading of machine-harvested tea leaves. The experimental results demonstrated that the machine achieved a screening rate exceeding 70%, with classification accuracy for high-quality tea and general tea exceeding 90%. Zhang et al.^4^ employed various machines, including air sorter, rotary screens, and roller screens, for grading a given batch of tea leaves. Notably, the custom-made roller screen machine exhibited superior grading efficacy. Additionally, the grading of high-quality tea leaves was further enhanced by employing an air sorter following the initial screening. Chen et al.^5^ addressed quality control during the air sorting process of tea leaves. The research was conducted on the efficiency and quality changes of tea leaf sorting at different wind speeds, ultimately determining the optimal wind speed. Liu^6^ delved into the automated sorting method for tea color sorter, employing convolutional neural networks (CNN) and machine vision. The color features were extracted by deep learning (DL) algorithm to classify the fresh tea leaves, resulting in a substantial improvement in the accuracy and efficiency of tea color sorters. Wu^7^ investigated non-destructive sorting methods for tea leaves by analyzing the optical characteristics of tea leaves. A tea leaf sorting system based on sensors was devised and achieved rapid and precise tea leaf sorting. Yan et al.^8^ leveraged image recognition technology to grade machine-harvested tea leaves by extracting the morphological features. Jiang^9^ addressed the challenge of low accuracy in grading machines by proposing a method for further grading tea leaves following an initial grading. The multiple texture features were extracted from grayscale and denoised tea leaves’ images, utilizing them as inputs to establish a least squares support vector machine (SVM) model. Experimental results validated the effectiveness of this model in achieving favorable grading outcomes. Zhang et al.^10^ employed a fusion approach to grade spring tea leaves, involving various preprocessing techniques to segment tea leaves from image backgrounds. Fourteen morphological and texture features extracted from the images were fused, and Histogram of Oriented Gradient (HOG) features were separately extracted. Classification models were constructed using both the fused features and the HOG features as inputs. Results indicated that the classification model using the fused features achieved the highest grading accuracy. Wang et al.^11^ accomplished rapid quality assessment of tea leaves by constructing a predictive model to forecast tea leaf composition. Borah et al.^12^ utilized wavelet transform to extract texture features from tea leaf images and created a classification model. This model exhibited superior accuracy compared to models utilizing texture features based on statistical moments. Laddi et al.^13^ pioneered a machine vision-based sensory quality evaluation model for grading tea leaves.

At present, machine sorting is widely used in tea production, but the problem of damage to fresh tea leaves and low grading accuracy is prominent. On the other hand, machine vision-based classification methods generally have low classification accuracy, and most models are focused on the grading of artificially scattered fresh tea leaves^14^. However, in the actual production process, a large number of fresh tea leaves are randomly stacked together for sorting. To the best knowledge of the authors, few research in the literature has study the classification methods for randomly stacked fresh tea leaves.

In light of these challenges, this paper introduces an innovative approach for assessing the quality of fresh tea leaves, employing a fusion of image recognition and a DL algorithm. This method relies on an integrated image acquisition system to capture high-quality tea leaf images, subsequently training and recognizing these images using suitable object detection models. Notably, this approach not only attains precise grading of orderly scattered tea leaves proportionally but also excels in grading randomly stacked tea leaves, closely mirroring real-world production requirements.

The purpose of the present paper is to achieve high-accuracy grading of dispersed fresh tea leaves in proportion, and it also achieves high-accuracy grading of flat fresh tea leaves that are closer to actual production. The highlights of this paper are summarized as follows:

1. Most of the fresh tea leaves picked are a mixture of different numbers of leaf buds, resulting in the blending of various grades of fresh tea leaves. Therefore, it is necessary to grade the fresh tea leaves before processing. This article focuses on the grading study of piled fresh tea leaves in actual processing plants, to the best knowledge of authors, no research in the literature has not been done before.
2. A method for determining the quality grades of tea fresh leaves based on the combination of image recognition and deep learning model has been proposed. This method can achieve high accuracy grading of tea fresh leaves by identifying the number of different leaf buds. The employed volov8 model has been optimized to improve recognition accuracy and computational efficiency. This high-accuracy grading method for tea fresh leaves is of great significance.

## Materials and methods

The used in this study were obtained the from the tea production region of Rizhao. The experiment was carried out at the MlE Research Center, College of Mechanical and Electronic Engineering, Shandong Agricultural University. The specific methods are detailed in the following sections.

This study complies with the IUCN Policy Statement on Research Involving Species at Risk of Extinction and the Convention on the Trade in Endangered Species of Wild Fauna and Flora. All aspects of his study were conducted in compliance with relevant institutional. national. and international guidelines and legislation.

### Data acquisition and processing

#### Data acquisition

**Figure 1:**
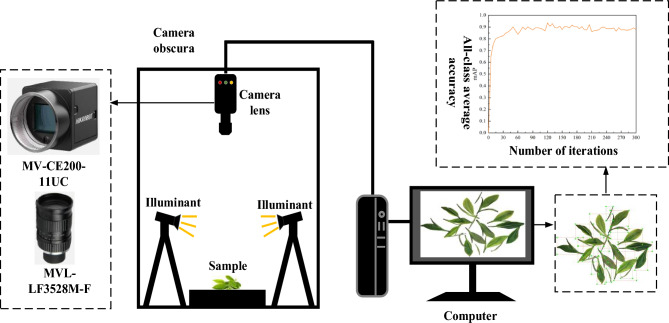
Image acquisition system.

**Figure 2:**
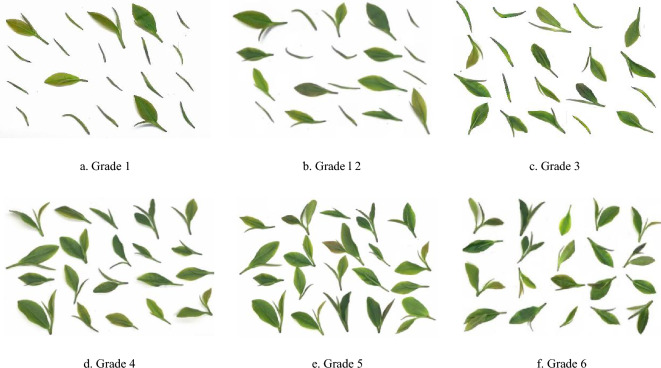
Graphical representation of grading of the orderly scattered fresh tea leaves.

**Figure 3:**
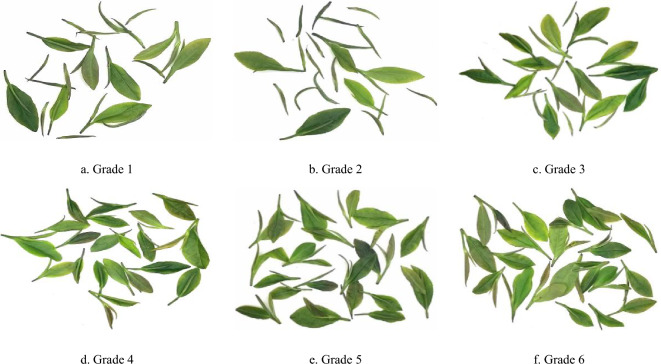
Graphical representation of grading of the randomly stacked fresh tea leaves.

China, as a prominent tea-producing nation, boasts vast tea cultivation areas spanning diverse regions. Among these, Rizhao City in Shandong Province stands out as one of the world's three leading coastal green tea hubs, and claims the title of the highest green tea producer in Shandong. Rizhao's green tea has garnered prestigious titles such as the "new aristocrat of Chinese green tea" and the "premier tea of the Jiangbei region." It serves as a representative of not only Shandong's green tea but also green tea produced across the entire country. (delete) Hence, this research focused on fresh green tea leaves sourced from Rizhao city in Shandong province, China^15^. In accordance with Rizhao City's local standards (DB37/T541-2005), the fresh tea leaves were categorized into six distinct grades based on the distinct bud and leaf combinations. The classification is conducted to distinguish the quantities of single bud, one bud one leaf, and one bud two leaves, in order to calculate the proportion of single bud for classification. The composition of these six grades of fresh leaves is detailed in Table 1. Subsequently, 100 images each of orderly scattered and randomly stacked tea leaves were captured for every grade. The image acquisition system was composed of a Sony IMX183 sensor, a 20-megapixel MV-CE200-11UC CMOS color industrial camera boasting a maximum frame rate of 19.2fps, and an MVL-LF3528M-F industrial lens featuring a 35 mm focal length, 0.40% optical distortion, and an F-Mount interface, as displayed in Fig. 1, while Figs. 2 and 3 showcase the orderly scattered and randomly stacked tea leaves, respectively.

**Table 1:** Grades of fresh tea leaves.

Fresh leaf grades	Fresh leaf composition
Grade 1	Composed of a single bud and one bud and one leaf, and a single bud > 70%
Grade 2	Composed of a single bud and one bud and one leaf, and 70% ≥ single bud > 40%
Grade 3	Composed of a single bud and one bud and one leaf, and one bud and one leaf ≥ 40%
Grade 4	Composed of one and two leaves of one bud, and one leaf of one bud ≥ 70%
Grade 5	Composed of one and two leaves of one bud, and 70% ≥ one leaf of one bud > 40%
Grade 6	Composed of one and two leaves of one bud, and two leaves of one bud ≥ 40%

#### Dataset construction

The performance of the classification models hinges significantly on the size of the training dataset. A small dataset can introduce issues like overfitting and reduced accuracy, highlighting the importance of dataset augmentation^16^. To bolster the model's robustness and enhance training outcomes, this study employed techniques like rotation, flipping, and contrast adjustment to augment the images of fresh tea leaves. The augmentation of the dataset's size and diversity is achieved through the application of random transformations to the original images. The rotation angle can be randomly chosen, for example, selecting a random angle between 0 and 360° for rotation. Flipping can be horizontal or vertical, or both horizontal and vertical at the same time. Contrast adjustment can be achieved by adjusting the brightness, contrast, or grayscale values of the image. This approach yielded a total of 700 distinct images for both orderly scattered and randomly stacked fresh tea leaves, resulting in 2800 images per category. To capture both the category and location details of tea leaves within the images, the Labelimg tool was utilized for annotation. Enhancement was performed on a total of 600 images across 6 Grades using three different methods, resulting in a total of 3600 enhanced images. The dataset comprised 4200 images, which were subsequently divided into training, validation, and testing sets. For the sake of efficient subsequent training, the dataset encompassing orderly scattered and randomly stacked fresh tea leaves was divided into training, validation, and testing sets, distributed in a 6:2:2 ratio.

### Methodology

#### YOLOv8x model

**Figure 4:**
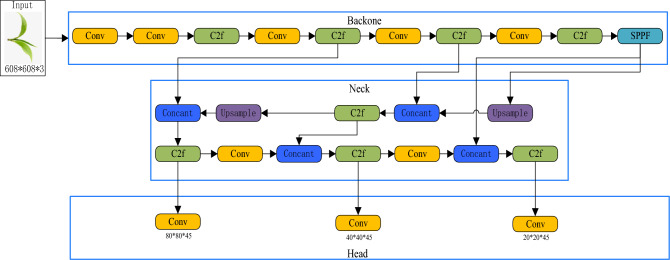
YOLOv8x network structure.

YOLOv8 is a one-stage object detection model that has evolved from YOLOv1 to YOLOv7. It offers four model frameworks: YOLOv8n, YOLOv8s, YOLOv8m, YOLOv8l and YOLOv8x, distinguished by variations in network depth and width^17^. Notably, YOLOv8x is the most substantial among them and delivers superior classification accuracy. The network structure of YOLOv8x, depicted in Fig. 4, comprises four main components: Input, Backbone, Neck, and Head.

**Figure 5:**
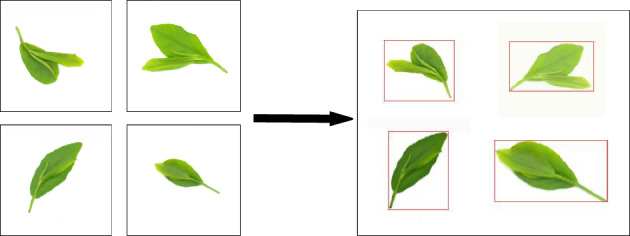
Mosaic enhanced rendering.

The input module integrates three modules: Mosaic data augmentation, Adaptive anchor box calculation, and Adaptive image scaling. Mosaic data augmentation combines multiple images to generate a novel image, thereby enhancing the model's capacity to detect small objects. The effectiveness of Mosaic augmentation is depicted in Fig. 5. Adaptive anchor box calculation automates the generation of initial anchor boxes^18^. Adaptive image scaling reduces black borders' size compared to conventional scaling and minimizes the inclusion of extraneous information.

The Backbone comprises two components: Focus and CSP, primarily responsible for feature extraction from the images. The neck section incorporates a feature pyramid and a path aggregation network to amalgamate features from different layers and detect objects of varying sizes. The head section is composed of convolutional layers, pooling layers, and fully connected layers, primarily tasked with object classification, regression, and generating output^19,20^.

#### Optimization of the YOLOv8x for fresh tea leaves’ classification.

##### Space pyramid pooling improvements

SPP (Spatial Pyramid Pooling) is a pooling structure used for image processing and computer vision tasks. It was introduced in 2014 by Kaiming He and others in the paper "Spatial Pyramid Pooling in Deep Convolutional Networks for Visual Recognition," and has been applied to image classification tasks. This module can perform standard pooling on images of different sizes and ultimately combine them into a column of features of the same size as input for fully connected layers^21^.

**Figure 6:**
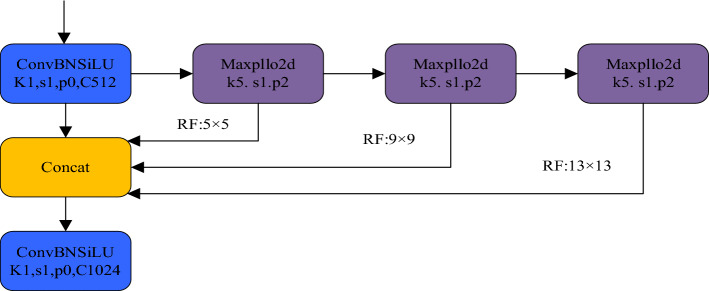
SPPF network structure.

**Figure 7:**
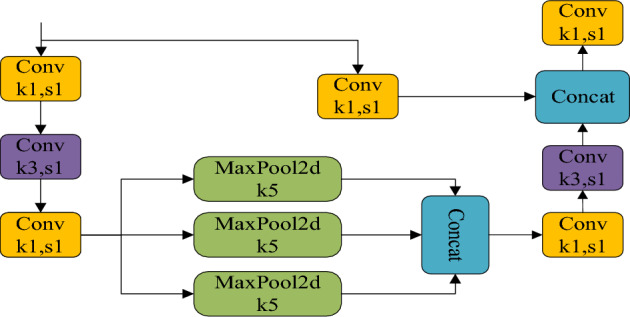
SPPCSPC network structure.

Considering that the target size of fresh tea leaves is relatively small, higher precision is required for the target detection network. In this study, the SPPCSPC module is used to replace the original SPPF module of YOLOv8 to improve the model. The SPPCSPC module is obtained by integrating the CSP (Cross Stage Partial) structure on the basis of the SPP module. In the SPPCSPC, the overall input is divided into two different branches. The middle 3 × 3 convolution is not grouped, and is still a standard convolution, while the right side is a point convolution. Finally, the information flow output by all branches is concatenated. The SPPCSPC has a significant improvement in the target detection network compared to the original SPP module and the SPPF module used in YOLOv8. It can play a better role for smaller targets such as fresh tea leaves. The structure of the SPPF module is shown in Fig. 6, and the structure of the SPPCSPC module is shown in Fig. 7. The SPPCSPC structure mainly consists of two sub-structures: the SPP structure and the CSPC (Cross Stage Partial Connections) structure. Its main idea is to introduce partial connections across stages into the network to replace the traditional serial connection method in convolutional neural networks for feature propagation, in order to solve the bottleneck problem of information transmission, improve feature propagation efficiency, and better utilize information between low-level and high-level features. The adoption of the SPPCSPC structure is beneficial for the recognition of fresh tea leaf targets, and the model has a better extraction effect on target features such as color and texture.

##### Concentration-based attention module (CBAM)

**Figure 8:**
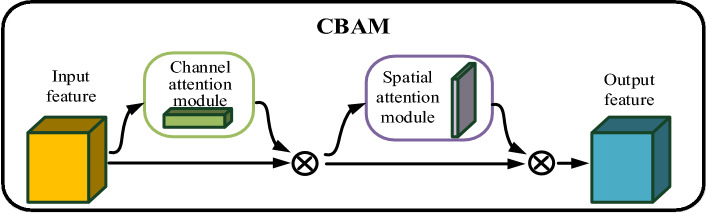
CBAM structure diagram.

The attention mechanism plays a crucial role in improving the model's focus on regions of interest within an image by assigning varying weights to different features^22^. For the fresh tea leaves’ images, this means giving more weight to the features of the fresh tea leaves and less weight to the white background. The CBAM structure is visually depicted in Fig. 8.

**Figure 9:**
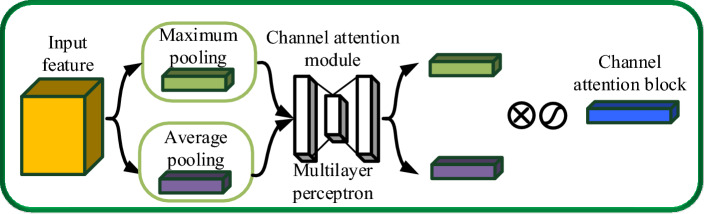
Realization process of channel attention mechanism.

The convolutional attention module comprises two main components: the channel attention module and the spatial attention module^23^. The channel attention mechanism functions by conducting max pooling and average pooling on the input feature maps in the spatial dimension, generating two weight vectors of size 1 × 1. These weight vectors are subsequently processed through a multi-layer perceptron with shared network parameters, which transforms the two weight channels. Finally, the two weight channels are merged and activated to produce the ultimate channel attention weights^24^. The process of implementing the channel attention mechanism is visually outlined in Fig. 9.

**Figure 10:**
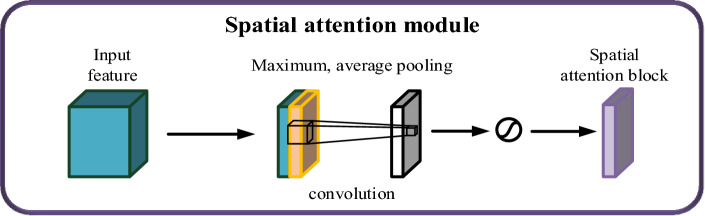
The realization process of the spatial attention mechanism.

The execution of the spatial attention mechanism entails the utilization of max pooling and average pooling on the input feature map of fresh tea leaves across the channel dimension. This yields two weight vectors with a channel size of 1 and dimensions H and W. These two weight vectors are subsequently stacked and subjected to a convolution operation, resulting in a weight vector with a channel size of 1 and dimensions H and W. Following this, the obtained feature map undergoes activation through a designated function to derive the spatial attention weights^25^. The process of implementing the spatial attention mechanism is visually depicted in Fig. 10.

**Figure 11:**
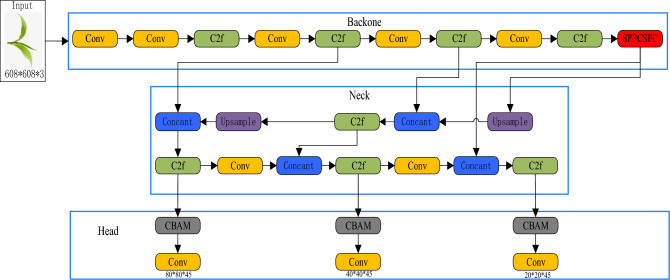
YOLOv8x-SPPCSPC-CBAM network structure diagram.

The YOLOv8x-SPPCSPC model undergoes enhancement through the integration of the CBAM module, culminating in the YOLOv8x-SPPCSPC-CBAM model. The structural representation of the YOLOv8x-SPPCSPC-CBAM configuration is displayed in Fig. 11.

## Model training and results analysis

In addition to training the tea fresh leaf dataset with the YOLOv8x-SPPCSPC-CBAM model, this study also employs three control models: Faster R-CNN, YOLOv5x and YOLOv8x. Training the tea fresh leaf dataset with these four models allows for a comprehensive comparison of their training results.

### Training parameter settings

The experimental setup utilized a Windows 11 operating system with a 64-bit architecture, an Intel(R) Core (TM) i9-1390000HX CPU @ 5.4 GHz, 16.00 GB of RAM, and an NVIDIA GeForce RTX 4060 GPU. The YOLOv8x-SPPCSPC-CBAM model was configured with an input size of 608 and a batch input size of 8. Initial learning rate settings included a value of 0.0032, a gradient descent momentum of 0.843, and a weight decay coefficient of 0.00036. To account for the increased complexity of classifying scattered tea leaves compared to stacked tea leaves, the training iterations for scattered tea leaves were set to 300, while the training iterations for stacked tea leaves were set to 400. In addition, due to the early stop mechanism in yolov8, which determines whether the model has reached the optimal performance by monitoring the model's performance metrics on the validation set, such as map (mean Average Precision), when the model's performance metrics on the validation set do not improve for a number of consecutive training rounds, the early stop mechanism is triggered to stop continuing the training to avoid overfitting. The current best performing model is merged and saved. Which the training iterations for scattered tea leaves were 253 and the training iterations for stacked tea leaves were 189.

### Results analysis

#### Evaluation indicators

The evaluation of the tea fresh leaf recognition and grading model in this study primarily relies on four key performance metrics: precision (P), recall (R), mean Average Precision (mAP) and Number of images processed per second(it/s)^16,17^. In this study, the labels for fresh tea leaves are categorized into three groups based on the number of leaf buds. It is assumed that positive represents the single bud category, while negative represents the non-single bud category. Therefore, TP denotes the quantity of instances predicted as single bud that are actually single bud, FP denotes the quantity of instances predicted as single bud that are actually non-single bud, FN denotes the quantity of instances predicted as non-single bud that are actually single bud, and TN denotes the quantity of instances predicted as non-single bud that are actually non-single bud. The formulas for calculating precision (P) and recall (R) are as follows:

$$P = \frac{TP}{{TP + FP}}\;\;\;R = \frac{TP}{{TP + FN}}$$

The precision P denotes the proportion of correct positive predictions among the total number of positive predictions, while the recall R represents the proportion of correct positive predictions among the total number of actual positive instances.

On a graph with precision P as the vertical axis and recall R as the horizontal axis, the average precision (AP) is defined as the area enclosed by the precision-recall (PR) curve and the two axes. The variable k denotes the number of categories assigned to an image during the process of labeling. The formula for calculating the mean average precision (mAP) across all classes is as follows:

$$mAP = \frac{{\sum\nolimits_{i = 1}^{k} {AP_{i} } }}{k}$$

The mean average precision (mAP) is a crucial evaluation metric for object detection models, calculated as the average precision across all classes.

While all these metrics provide valuable insights into the model's performance, mAP is regarded as the most comprehensive assessment tool, offering a holistic overview of the model's effectiveness. Nonetheless, precision and recall are also considered for their individual contributions, with mAP taking precedence as the primary evaluation metric. (delete).

#### Comparison of experimental results of different models

**Figure 12:**
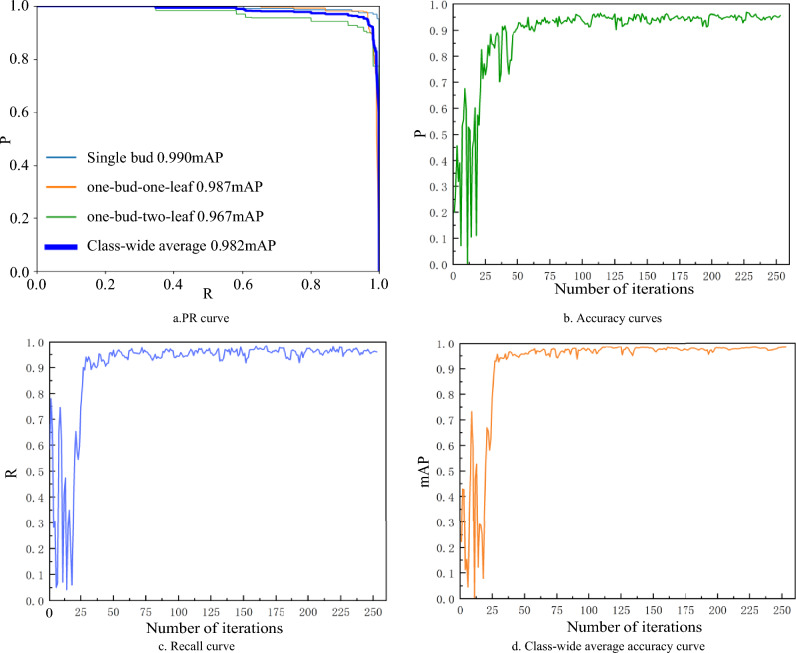
YOLOv8x-SPPCSPC-CBAM training results on scattered fresh tea leaves diagrams.

The training of the scattered fresh tea leaves dataset was conducted using the YOLOv8x-SPPCSPC-CBAM model, and the resulting training outcomes are presented in Fig. 12.

Based on Fig. 12, it's evident that the mean Average Precision value (mAP) remains consistently high, approaching 1, as the recall value (R) also approaches 1, with no significant decline. Furthermore, the values of P, R, and mAP show an overall upward trend during the initial iterations, and by the 100th iteration, all three metrics have reached a relatively stable phase.

Table 2 presents a comparison of the training results between the YOLOv8x-SPPCSPC-CBAM model and the Faster R-CNN, YOLOv5x and YOLOv8x models when trained on scattered fresh tea leaves.

**Table 2:** Training results of scattered fresh tea leaves.

Models	Precision P	Recall rate R	Average Precision Mean	it/s
Faster R-CNN	0.727	0.941	0.935	1.81
YOLOv5x	0.953	0.954	0.963	2.09
YOLOv8x	0.961	0.959	0.975	2.33
YOLOv8x-SPPCSPC-CBAM	0.958	0.967	0.982	2.77

According to the results in Table 2, the YOLOv8x-SPPCSPC-CBAM model achieves a precision (P) value of 0.958, a recall (R) value of 0.967, mean average precision (mAP) value of 0.982 and Number of images processed per second(it/s) value of 2.77. Although the P value of YOLOv8x-SPPCSPC-CBAM is slightly lower (0.003) than that of YOLOv8x, it surpasses all other models in terms of R, mAP and it/s values. In comparison to Faster R-CNN, YOLOv5x and YOLOv8x, the YOLOv8x-SPPCSPC-CBAM model demonstrates an improvement of 0.026, 0.013, and 0.008 in the R value, and an improvement of 0.047, 0.019, and 0.007 in the mAP value, and an improvement of 0.96, 0.58, and 0.44 in the it/s value, respectively. Therefore, the YOLOv8x-SPPCSPC-CBAM model exhibits superior performance in the recognition and classification of scattered fresh tea leaves.

**Figure 13:**
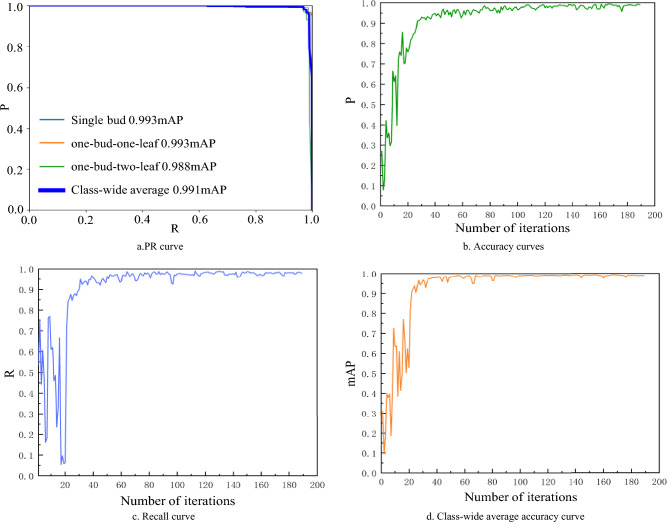
YOLOv8x-SPPCSPC-CBAM training results on stacked fresh tea leaves diagrams.

The training results of the YOLOv8x-SPPCSPC-CBAM model on the stacked fresh tea leaves are displayed in Fig. 13.

It can be observed that among the three categories from Fig. 13a, the PR curve for one bud and one leaf encloses the smallest area bounded by the horizontal and vertical axes, while the PR curve for one bud and two leaf has the largest enclosed area. Notably, after approximately 115 iterations, the precision rate (P) stabilizes. It takes until around the 85th iteration for mAP to reach a relatively stable stage.

The training results of the Faster R-CNN, YOLOv5x, YOLOv8x and YOLOv8x-SPPCSPC-CBAM models on the dataset of stacked fresh tea leaves are summarized in Table 3.

**Table 3:** Training data of stacked fresh tea leaves.

Models	Precision P	Recall rate R	Average Precision Mean	it/s
Faster R-CNN	0.757	0.957	0.929	1.52
YOLOv5x	0.960	0.964	0.967	1.78
YOLOv8x	0.982	0.973	0.986	2.13
YOLOv8x-SPPCSPC-CBAM	0.991	0.977	0.991	2.35

According to Table 3, the YOLOv8x model achieved a precision (P) value of 0.982, a recall (R) value of 0.973, and a mean average precision (mAP) value of 0.986 for the classification of stacked tea leaves. These values are notably higher than those of the Faster R-CNN model, with a difference of 0.225 for P, 0.016 for R, 0.057 for mAP, and 0.61 for it/s. In comparison to the YOLOv5x model, the YOLOv8x model also outperforms it, with higher P, R, mAP and it/s values by 0.022, 0.009, 0.019 and 0.35, respectively. Hence, the YOLOv8x model exhibits superior recognition performance for flat tea leaves when compared to both the Faster R-CNN and YOLOv5x models.

Furthermore, the YOLOv8x-SPPCSPC-CBAM model demonstrates even better results in four key performance metrics for stacked tea leaves when compared to the YOLOv8x model. It shows an increase of 0.009 for precision (P), 0.004 for recall (R), 0.005 for mAP and 0.22 for it/s. Consequently, among the four models evaluated, the YOLOv8x-SPPCSPC-CBAM model stands out as the top performer in classifying the stacked fresh tea leaves.

The comparison of Tables 2 and 3 indeed highlights the superior training performance of the YOLOv8x-SPPCSPC-CBAM model, not only for scattered tea leaves but also for stacked tea leaves. This shows the versatility and effectiveness of the YOLOv8x-SPPCSPC-CBAM model in classifying both scattered and stacked tea leaves.

### Fresh tea leaf identification and grading

In the post-processing stage of the model recognition's detection component, code is implemented to calculate the count and proportion of identification boxes for each category. This process yields three distinct categories: single bud, one bud and one leaf, and one bud and two leaves.

To directly determine the quantity of single buds, one bud and one leaf, or one bud and two leaves in the identified images, we have enhanced the classification model in this study. The improved classification model can display the count of single buds, one bud and one leaf, or one bud and two leaves in the top-left corner of the image. By utilizing the count of fresh tea leaves from a particular category obtained in this manner, the proportion of that specific type of fresh tea leaves in the image can be calculated. Subsequently, the tea leaves can be graded based on this proportion. For instance, in the case of single buds, the proportion calculation is as follows:

1 $$P_{N} = \frac{N}{N + M + L} \times 100\%$$

In the Eq. (1), *P*_*N*_ represents the proportion of single buds among all the fresh tea leaves captured in the images. *N* denotes the count of identified single buds, *M* represents the count of identified one bud and one leaf, and* L* represents the count of identified one bud and two leaves.

**Figure 14:**
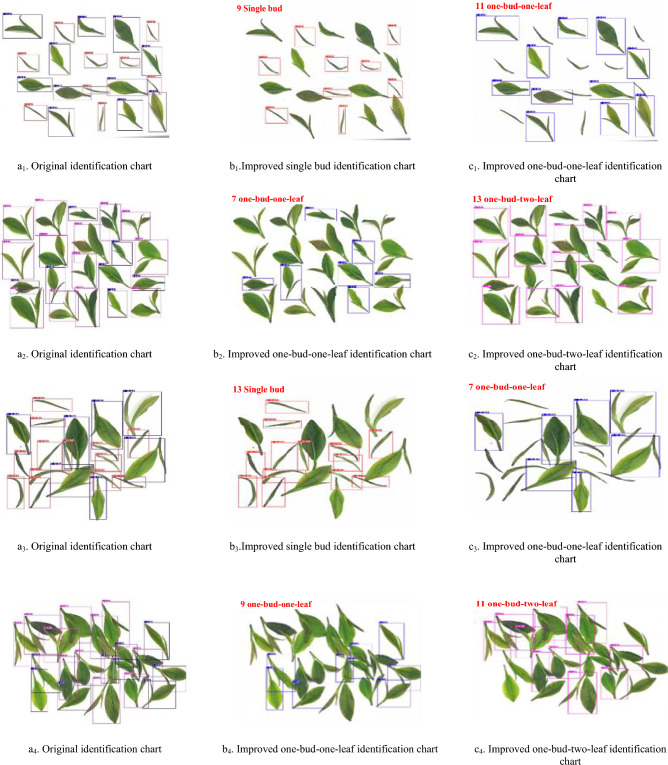
YOLOv8x-SPPCSPC-CBAM Classified images.

The original classification model and the improved classification model, YOLOv8x-SPPCSPC-CBAM, were employed to identify the test set images. The results for identifying scattered fresh tea leaves and stacked fresh tea leaves are presented in Tables 4 and 5, respectively. The classification results in the images are shown in Fig. 14.

**Table 4:** Classification results of scattered fresh tea leaves.

Grade	Number of images	Number of correct images identified
Grade 1	92	88
Grade 2	96	92
Grade 3	95	91
Grade 4	93	90
Grade 5	94	90
Grade 6	90	87

**Table 5:** Classification results of stacked fresh tea leaves.

Grade	Number of images	Number of correct images identified
Grade 1	91	90
Grade 2	94	93
Grade 3	93	93
Grade 4	93	93
Grade 5	96	95
Grade 6	93	92

## Conclusion

1. This study introduces a novel approach to determining the quality grades of fresh tea leaves by merging image recognition with deep learning algorithms. It leverages a dedicated hardware system to capture high-quality images of fresh tea leaves, employs a DL algorithm for tea leaves and detection, and enhances the classification model to enable the grading of fresh tea leaves. The classification results validate that this proposed model (YOLOv8x-SPPCSPC-CBAM) satisfactorily meets the stringent accuracy requirements for grading fresh tea leaves.
2. In this research, the YOLOv8x model was enhanced by integrating the Space pyramid pooling improvements (SPPCSPC) for size detection and the concentration-based attention module (CBAM). A comprehensive comparison was performed between the improved YOLOv8x model and other popular models, including Faster R-CNN, YOLOv5x, and YOLOv8x. The outcomes indicate that the proposed YOLOv8x-SPPCSPC-CBAM model exhibited remarkable classification capabilities, excelling in identifying scattered and stacked fresh tea leaves.

Due to the wide variety of tea, the generalization of the proposed method to more tea varieties would conducted in our future work.

## Data Availability

The raw and processed data required to reproduce these results are available by contacting the author—Yu’xiang He (1157683420@qq.com).
